# CATEcor: An Open Science, Shaded-Truss, Externally-Occulted Coronagraph

**DOI:** 10.1007/s11207-024-02314-x

**Published:** 2024-06-10

**Authors:** Craig DeForest, Daniel B. Seaton, Amir Caspi, Matt Beasley, Sarah J. Davis, Nicholas F. Erickson, Sarah A. Kovac, Ritesh Patel, Anna Tosolini, Matthew J. West

**Affiliations:** 1 Southwest Research Institute, Boulder, CO USA; 2 Independent Researcher, Boulder, CO USA

**Keywords:** Solar eclipse, Solar K corona, Solar instrumentation

## Abstract

We present the design of a portable coronagraph, CATEcor (where CATE stands for Continental-America Telescope Eclipse), that incorporates a novel “shaded-truss” style of external occultation and serves as a proof-of-concept for that family of coronagraphs. The shaded-truss design style has the potential for broad application in various scientific settings. We conceived CATEcor itself as a simple instrument to observe the corona during the darker skies available during a partial solar eclipse, or for students or interested amateurs to detect the corona under ideal noneclipsed conditions. CATEcor is therefore optimized for simplicity and accessibility to the public. It is implemented using an existing dioptric telescope and an adapter rig that mounts in front of the objective lens, restricting the telescope aperture and providing external occultation. The adapter rig, including occulter, is fabricated using fusion deposition modeling (FDM; colloquially “3D printing”), greatly reducing cost. The structure is designed to be integrated with moderate care and may be replicated in a university or amateur setting. While CATEcor is a simple demonstration unit, the design concept, process, and trades are useful for other more sophisticated coronagraphs in the same general family, which might operate under normal daytime skies outside the annular-eclipse conditions used for CATEcor.

## Introduction

**Figure 1:**
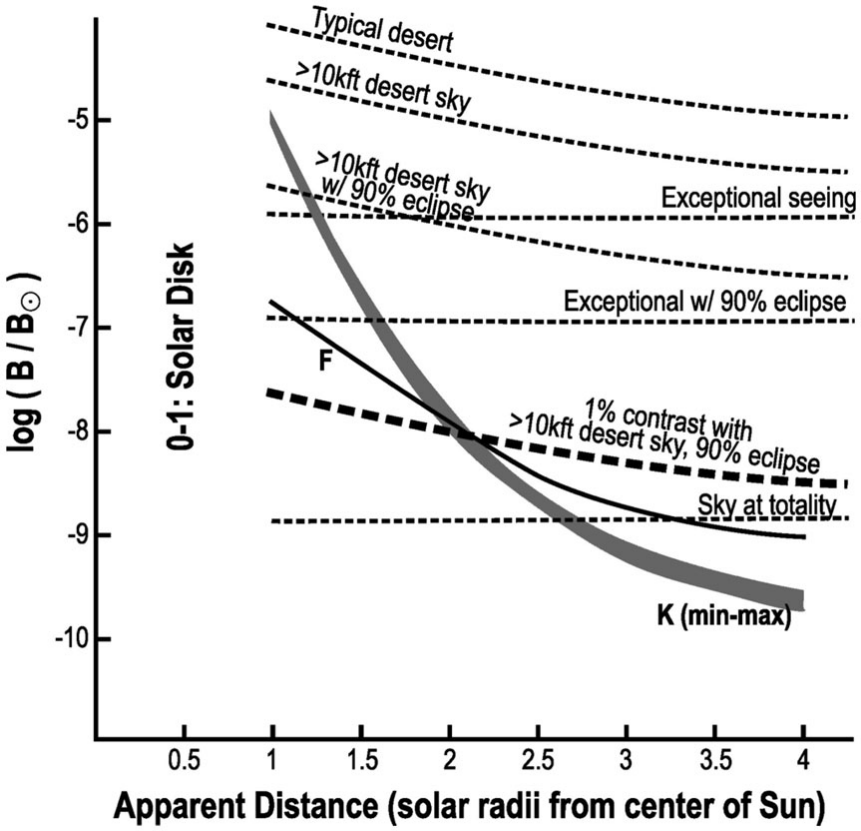
Ground-based solar coronagraphs must contend with not only instrumental scatter but the background sky brightness (dotted lines for various conditions), which falls off slowly compared with the brightness of the K-corona itself (grey line). This drives coronagraph solutions that are optimized for the very low corona, requiring internal occultation. In very high-altitude conditions or during an annular solar eclipse, the sky brightness is reduced by an order of magnitude or more, enabling externally-occulted coronagraphs to image the corona to 3 or more solar radii. Figure adapted with permission from Golub and Pasachoff (2009).

Solar coronagraphs work by blocking out the Sun to produce an “artificial eclipse” (Lyot, 1930), allowing imaging of the corona itself around the bright Sun. Ground-based instruments must contend not only with instrumental scattering but also with sky brightness (Figure 1). Such instruments are optimized to image the innermost portion of the corona, which rises above the local sky brightness; they include the Mauna Loa K-coronameter (Altschuler and Perry, 1972; Fisher et al., 1981) and more modern instruments including the Large Angle and Spectroscopic Coronagraph/C3 (LASCO/C3: Tomczyk and McIntosh, 2009) and K-Cor (de Wijn et al., 2012). Instruments that image low coronal altitudes generally use internal occultation, in which an initial focusing optic produces a real image of the Sun, and the light in that image is rejected from the instrument by a physical object or a hole in a reflecting optic. Internal occultation allows very precise selection of which altitudes within the corona will be imaged, at the cost of requiring a very-low-scatter initial optic and generating instrumental stray light in the far field.

Spaceborne coronagraphs have practically no limitation from sky brightness, and can image the corona much farther from the Sun, by using external occultation to reject the bulk of the Sun’s rays even before the first optic. This greatly reduces stray light in the far field, at the cost of vignetting the field near the Sun itself. Examples of externally-occulted coronagraphs include the Large Angle and Spectroscopic Coronagraph/C3 (LASCO/C3: Brueckner et al., 1995) and the Solar Terrestrial RElations Observatory (STEREO/COR2: Howard et al., 2008). The most recent generation of spaceborne coronagraphs, including the Compact Coronagraph (CCOR: Thernisien et al., 2021) and the Polarimeter to Unify the Corona and Heliosphere/Narrow Field Imager (PUNCH/NFI: Colaninno et al., 2019), eschew internal or secondary occultation altogether and rely entirely on a highly engineered external occulter to reduce stray light to levels compatible with coronal imaging as far as 30 apparent solar radii ($\mathrm{R}_{\odot}$) from the Sun.

The limitations of ground-based coronagraphs are greatly mitigated during conditions that significantly improve (reduce) sky brightness. Figure 1 shows typical sky brightness curves under various conditions. During an annular solar eclipse, or at exceptionally high altitude in the atmosphere (e.g., above 80 000 ft. altitude), sky brightness can be reduced by an order of magnitude or more compared to high desert conditions; in turn, that may enable imaging of the middle corona (West et al., 2023) at or above 3 R_⊙_ from the Sun without flying an instrument into space or requiring a total solar eclipse. This insight led us to consider new instrument designs that might enable ground-based imaging of the middle corona, farther from the Sun than is possible from the ground under normal conditions.

The imaging quality required to capture the middle corona is modest by the standards of either spaceborne coronagraphs (which are optimized for very low stray light and small instrument dimensions) or ground-based coronagraphs (which are optimized to image very close to the Sun). Hence we did not consider a complete new instrument, but a module that could extend an existing, well-defined imaging system. We adopted the telescope and camera from the Citizen Continental-America Telescopic Eclipse 2024 (CATE24) program (Caspi et al., 2023; Patel et al., 2023), which in turn is a next-generation version of the original Citizen CATE (Penn et al., 2020) project carried out in 2017. The CATE24 observing equipment comprises a Long Perng doublet refracting telescope with 80 mm aperture and 500 mm focal length (f/6.25) on a German Equatorial Mount, and a FLIR Blackfly polarizing CMOS camera with an integrated multiplex polarizer mask, allowing recovery of Stokes I, Q, and U across the entire field of view (FOV) from each exposure. The imaging resolution is 2.9^′′^ FWHM, limited by diffraction from the aperture, and is matched to the 1.43^′′^/pixel plate scale of the overall system; see Patel et al. (2023) for further details.

CATEcor modifies the CATE24 telescope with a clip-on assembly comprising an aperture stop just in front of the telescope objective, and an external occulter 75 cm in front of the stop. The occulter is supported by a truss that remains fully shaded by the occulter itself; this reduces scatter from the support structure, and reduces or eliminates the need for a dark “vestibule” to support a pylon and the occulter, as in existing externally-occulted instruments (e.g. Howard et al., 2008). CATEcor thus embodies a new type of instrument, a “shaded-truss externally-occulted coronagraph^1^”. Compared to conventional internally-occulted coronagraph designs, the shaded-truss approach has lighter weight, lower instrumental stray light in the far field, and the beneficial smooth inner-field sensitivity rolloff that is characteristic of other externally-occulted designs, at the cost of a more complex vignetting function (as the instrument looks *through* a complex, structured truss).

The entire CATEcor assembly may be integrated from readily obtainable parts and 3D printed elements, permitting individuals to reproduce CATEcor for hobbyist or student applications; thus we refer to CATEcor as an “open science” instrument.

In the following sections, we describe the trade space and requirements that drive CATEcor, and present the actual design in sufficient detail to reproduce the instrument. Section 2 gives requirements for CATEcor; Section 3 presents the design concept, develops specifications for the optomechanical elements, and presents the design elements. Section 4 describes fabrication and integration steps for the instrument. Section 5 includes results from a full-Sun test observation. Section 6 contains discussion of the novel design space of CATEcor and its relevance to future instrumentation, and we draw conclusions in Section 7. We also used CATEcor to image the corona at an annular eclipse; that observation is detailed by Seaton et al. (2024).

## Requirements for CATEcor

Two objectives drove us to build CATEcor: (1) to demonstrate coronal imaging with at-hand materials including a low-cost commercial telescope available to amateurs (the CATE24 telescope; Patel et al., 2023) and supplies and manufacturing technology available to hobbyists; and (2) to validate a novel, simplified coronagraph concept for coronal imaging. The primary design requirements were:

Overall structure. CATEcor is intended as an easily manufacturable addition to the existing CATE24 telescopes, which may be directly mounted on the telescope and balanced with existing CATE24 project mounts, tripods, counterweights, etc. Alignment and calibration must be accessible to amateurs or students with the same skill level required of CATE24 operators in the main program.

Imaging resolution. The resolution requirement is driven by feature size in the corona. Streamer tops are approximately 2′ – 3′ across, levying an azimuthal-direction resolution requirement of 2′ at apparent distances of 1.8 R_⊙_ or more from disk center. This is modest or trivial for a small refractor such as the 80 mm aperture CATE24 telescopes, but significant for an externally-occulted coronagraph where the effective aperture may be very small, and drives geometric aspects of the shaded-truss design. A 2′ diffraction limit requires an effective aperture roughly 1 mm across or more. It also drives noise performance of the instrument, as in DeForest et al. (2018).

Field of view. CATEcor must be in principle able to image full circle from 1.5 R_⊙_ to 2.5 R_⊙_ under the dark sky associated with a 90% annular eclipse, and must capture sufficient altitude range of the corona to unambiguously demonstrate imaging. The CATE24 telescopes have adequate FOV, extending to at least 3 R_⊙_ in all directions. For an externally-occulted design, the inner edge of the FOV is set by the occulter/aperture geometry. The outer limit of the effective FOV is determined by background brightness and associated noise characteristics. CATEcor is expected to capture from close to 1.5 R_⊙_ to roughly 2 R_⊙_ with the possibility of a full solar radius from 1.5 – 2.5 R_⊙_, based on typical K-corona brightness (Figure 1).

## The CATEcor Design

**Figure 2:**
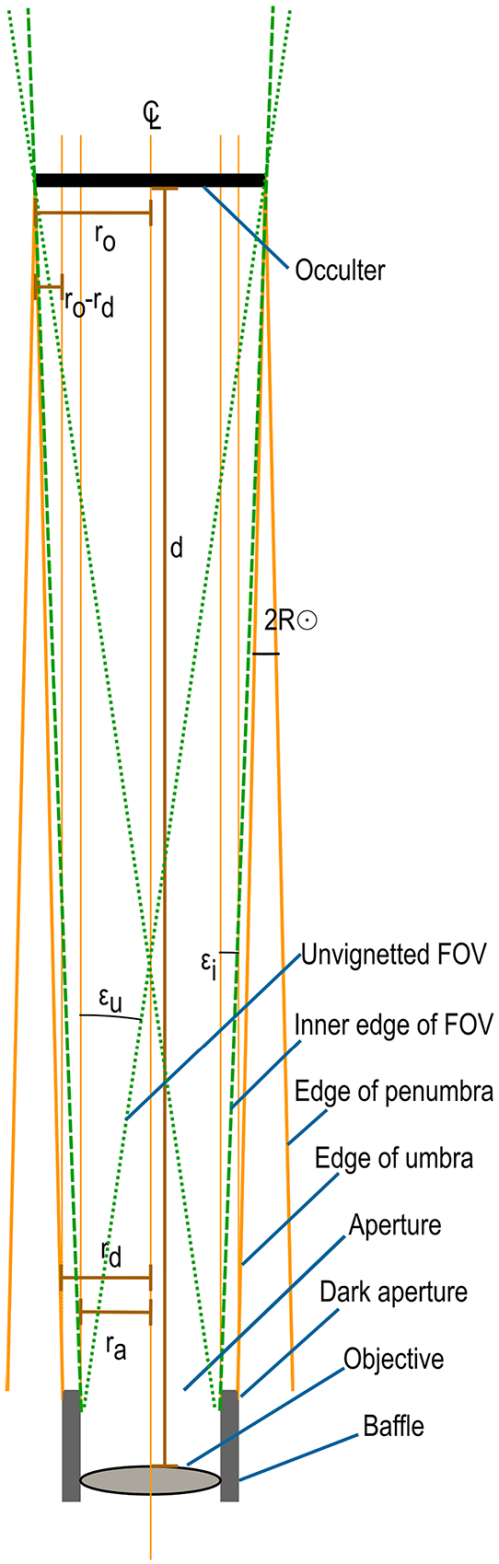
Geometry of a generic externally-occulted coronagraph shows the related geometrical quantities used to specify even the simple CATEcor instrument. The occulter is sized to completely shadow the aperture and the dark baffle area. The inner edge of the FOV, $\varepsilon _{i}$, is set by the angle between the edge of the occulter and the near edge of the aperture. The innermost unvignetted portion of the FOV, $\varepsilon _{u}$, is set by the angle between the edge of the occulter and the farthest edge of the aperture. The umbra and penumbra extend inward and outward, respectively, from the edge of the occulter as shown. The spreading angle between the umbral and penumbral boundaries is the apparent solar diameter 2 $\mathrm{R}_{\odot}$, and is exaggerated by a factor of 5 in this conceptual diagram.

We conceived CATEcor as a front-end external occulter-and-aperture assembly (Figure 2) to mount directly to the existing CATE24 telescopes, which are 80 mm diameter aperture tube-based dioptric telescopes with 100 mm diameter retractable dust covers. In keeping with the CATE24 project philosophy of reducing barriers to scientific measurement, we specifically designed CATEcor to be easily assembled from readily available hardware and hobbyist Fusion Deposition Modeling (FDM) equipment (“3D printing”). In principle, CATEcor could be duplicated by anyone with access to an amateur telescope, a computer, a 3D printer, and a hardware store.

**Figure 3:**
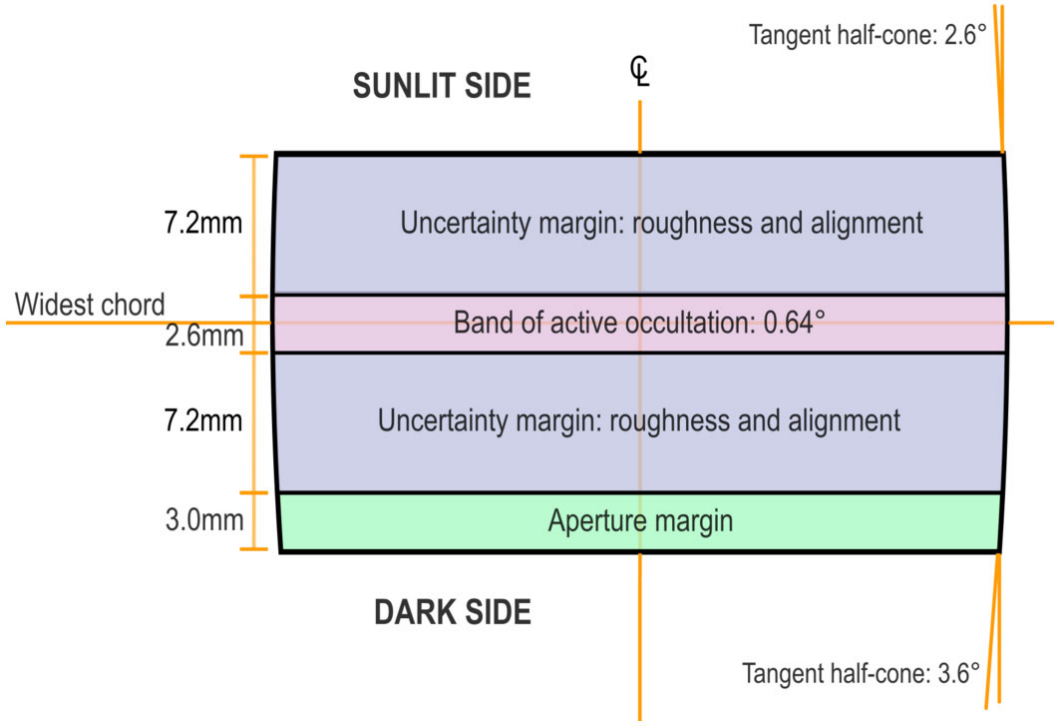
Conceptual cross-section of the CATEcor occulter shows the truncated ellipsoid, which approximates a shape with constant major radius of curvature (i.e., an ogive). The occulter is a figure of revolution about the centerline. The active occultation band obscures 2.4 $R_{\odot}$ of sky to block both the Sun’s disk and the 0.4 $R_{\odot}$ design margin. The additional thickness provides rigidity, mount holes for the occulted hexapod truss, and wide alignment tolerance of roughly $\pm 1^{\circ }$. Not shown: center through-hole for alignment and six dark-side blind holes for the hexapod truss.

**Figure 4:**
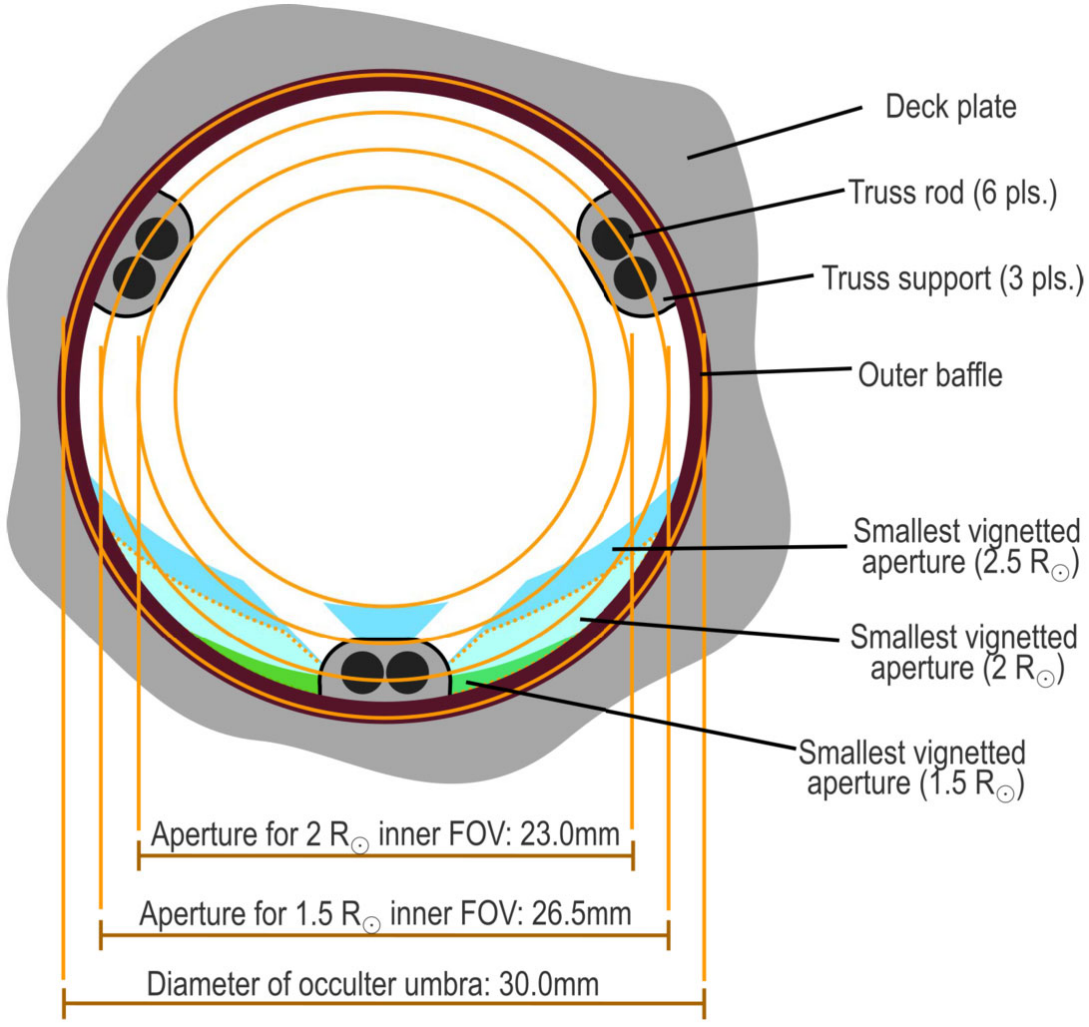
The CATEcor aperture, seen here from the point of view of the occulter, is sized to fit well inside the umbra of the occulter while still supporting imaging at 1.5 R_⊙_. A surrounding deck encloses a precise iris aperture (not shown). With ideal pointing the umbra of the occulter forms a 30 mm diameter circle; the baffle is entirely inside the umbra. The aperture is encroached upon by three small decks above the iris, supporting the six rods of the hexapod truss. The decks partially vignette the innermost portion of the FOV. The effective aperture is shown for three points on the sky, aligned with one of the hexapod supports: 1.5 R_⊙_, 2 R_⊙_, and 2.5 R_⊙_ from Sun center.

**Figure 5:**
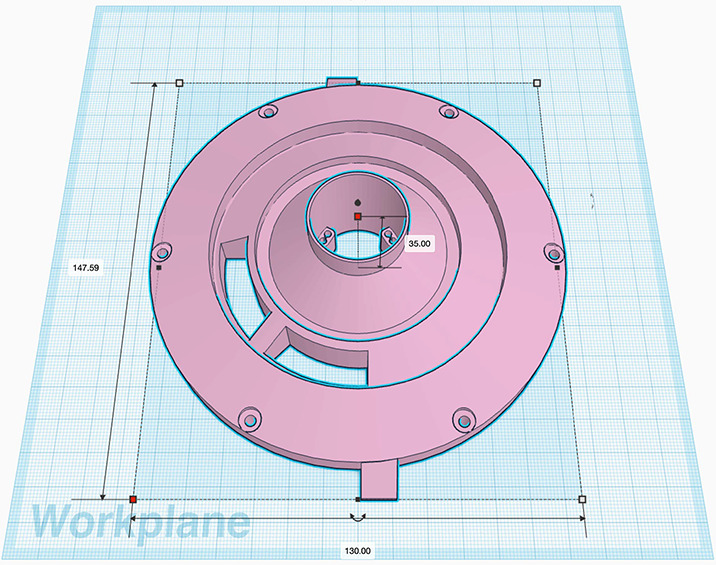
CATEcor aperture piece mounts to the CATEcor front plate, and is secured by six M3 bolts around the perimeter. Lateral stability is ensured by positive lock between the tabs on the front plate and matching recess holes on the underside of the aperture piece; 1/4 of the perimeter has a slot to allow adjustment of the adjustable-iris aperture stop in the interior. Small finger tabs on the perimeter help to mate/demate the press-fit features with the front plate. Two of the three hexapod mounting decks can be seen at the base of the central aperture.

**Figure 6:**
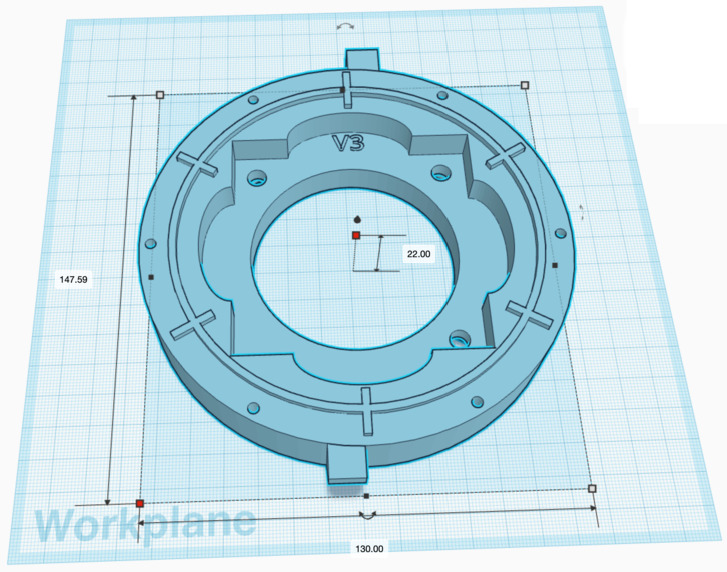
CATEcor front plate rests on top of the tube adapter. A circular groove at the bottom mates with the circular ridge at the top of the tube adapter. A central 55 mm diameter hole allows light to enter the telescope objective lens. A square recess in the structure accepts an adjustable-iris aperture stop (not shown). Radial ridges mate positively with radial recesses on the underside of the aperture piece, to ensure dimensional stability. Hexagonal recesses (not visible) on the underside mate with M6 through-bolts to secure the adjustable-iris aperture stop. The top and bottom feature “optical maze” mounting rings both for alignment and to prevent stray light entering through joints in the assembly. Small finger tabs on the perimeter help to mate/demate the press-fit alignment features with the aperture piece.

**Figure 7:**
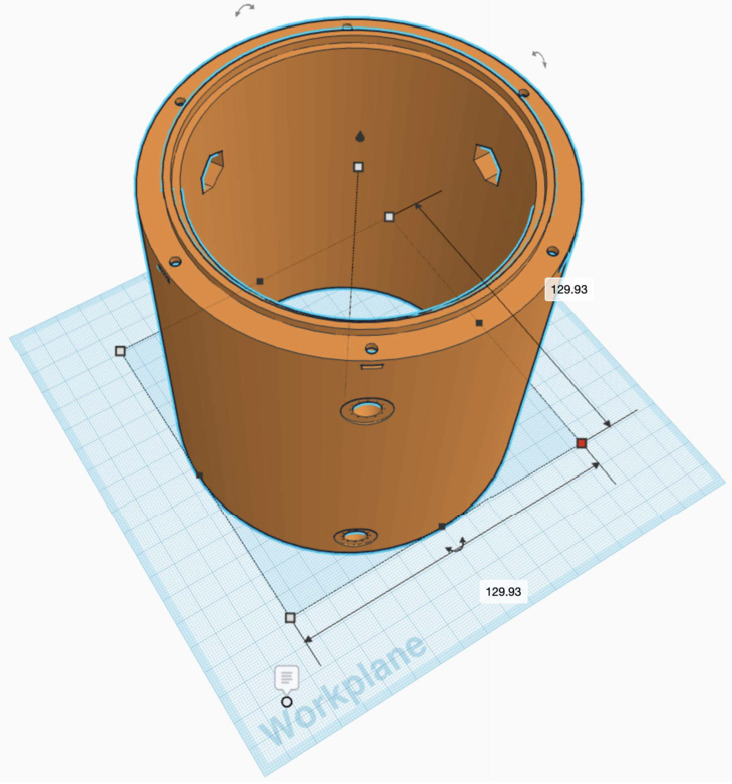
CATEcor tube extension is designed to slip over the dust cover of a CATE24 telescope. It is secured by six nylon M8 bolts with captive hex nuts on the inside and nylon washers and hex nuts on the outside. A circular mount ring aligns the tube and prevents external light from entering. The front plate and aperture are secured by six M3 through-bolts into captive square nuts.

The CATEcor assembly comprises fasteners and ancillary elements, linking four 3D printed parts: an external occulter (Figure 3), an aperture piece (Figures 4 and 5), a front plate (Figure 6), and a telescope tube extension (Figure 7). The occulter is attached to the aperture piece by a 75 cm carbon-fiber hexapod truss made from commercially available carbon-fiber rods, which are glued into 3D printed features in the occulter and aperture piece.

The telescope tube extension, front plate, and aperture piece were all designed using the extremely simple web-based tool TinkerCAD (Backman and Mononen, 2013). TinkerCAD was ideal because it is specifically designed to lower barriers to computer-aided design (CAD) by hobbyists and students, highlighting the accessibility of appropriate mechanical design tools for everyone. The largest drawback of TinkerCAD for this application is that it uses a coarse polyhedral tessellation for all curved surfaces. Typical outside dihedral angles produced by TinkerCAD, when approximating round surfaces, are 6^∘^ or more. This was acceptable for the mechanical interface structures such as the telescope attachment, but unacceptable for the occulter itself. We therefore used the open-source FreeCAD software (Riegel, Mayer, and contributors, 2002) for the occulter itself.

All of the designs of the 3D printed parts are freely available in “.stl” and other formats, and are available for download (DeForest, 2023).

In the following subsections, we describe and develop the fundamental design concept of a shaded-truss externally-occulted coronagraph (Section 3.1); develop the occulter design (Section 3.2); and describe the designs for the truss (Section 3.3), aperture (Section 3.4), front plate (Section 3.5), and telescope extension tube (Section 3.6).

### Shaded-Truss Externally Occulted Coronagraph Concept Design

The coronagraph design space is dominated by the observed strong radial gradient in the K-coronal brightness (Figure 1). Coronagraphs generally capture an annular FOV. The inner edge is limited by the geometry and diffraction characteristics of the occulter. The outer edge is limited by background sources of light, sky brightness for ground-based instruments or instrumental stray light for spaceborne instruments. At large apparent distances (elongations), the desired K-corona signal drops well below other background sources including the F-corona; but digital postprocessing enables separation of the K signal even at very large elongation angles and contrast ratios as low as a few × $10^{-4}$ (Jackson, 1985; DeForest, Howard, and Tappin, 2011), enabling both wide-field coronagraphs (e.g. Brueckner et al., 1995) and the relatively new field of heliospheric imaging (e.g. Eyles et al., 2003; Howard et al., 2008; DeForest et al., 2022). Although Fresnel diffraction is important to certain parts of coronagraph design (specifically the occulter), ray optics are sufficient to design most of the instrument geometry, with recourse to wave optics only for the detailed design of the occulter itself.

Externally-occulted coronagraphs trade simplicity (of a direct occulter casting a shadow on the entrance to a camera or telescope) for fuzziness of the boundary of occultation on the image plane. The fuzziness arises because external occulters at finite distance in front of the optics are imaged out of focus in the field of view. That is because the corona itself is at optically infinite distance, and therefore focusing the optics to image the corona necessarily defocuses the image of the much-closer occulter.

Figure 2 illustrates the basic geometry of a generic dioptric externally-occulted coronagraph, exaggerating the apparent size of the Sun by a factor of 5 for clarity. An occulter casts a shadow down the length of the instrument. Penumbral and umbral edges formed by the outer edge of the occulter are marked in orange. The occulter is sized such that the edge of the umbra lands outside a “dark aperture” that extends beyond the objective lens of the system. The principal design quantities are: $d$, the distance between the aperture and last effective plane of the occulter; $r_{a}$, the radius of the aperture; $r_{d}$, the radius of the dark zone behind the occulter; and $r_{o}$, the radius of the final disk of the occulter itself. While instrument dimensions are lengths, the extent of the annular field of view (FOV) is best described with solar elongation angles (apparent radial distances), $\varepsilon $. The inner edge of the FOV is set by the angle between the outermost edge of the occulter and the nearest portion of the active aperture of the imaging optics, i.e.,

1 $$ \varepsilon _{i} = \left ( r_{o} - r_{a} \right ) / d \,, $$

under the small-angle approximation that $\sin (\theta )=\theta $. Meanwhile, the first nonvignetted elongation $\varepsilon _{u}$ is just

2 $$ \varepsilon _{u} = \left ( 2r_{o} + 2r_{a} \right ) / d \,. $$

In turn, the occulter size $r_{o}$ is determined by the dark radius $r_{d}$, the distance $d$, and the apparent size of the Sun $R_{\odot}$. For very long instruments in which $r_{d} \ll r_{o}$, $r_{a}$ is negligible and $\varepsilon _{u} \ge 2~R_{\odot}$; for short instruments such as CATEcor, $r_{o} \approx r_{d}$ and $\varepsilon _{u} \ge 4~R_{\odot}$. CATEcor observations are vignetted throughout the anticipated effective FOV.

Note that $R_{\odot}$ varies between the dates of perihelion and aphelion. At the time of CATEcor’s initial deployment, in October, 2023, $R_{\odot}$ was roughly 16′ and therefore,

3 $$ r_{o} \geq r_{d} + R_{\odot }d = r_{d} + 4.654\times 10^{-3} d \,. $$

The actual diameter of the occulter is generally slightly larger than the equality of Equation 3, to allow for pointing error and/or a margin for Fresnel diffraction; in general, one can hold pointing margin in $r_{d}$ and use the equality rather than the inequality in Equation 3.

The inner edge of the FOV is the angle where the vignetting function reaches 0%, i.e., the angle at which a single ray can pass the occulter and enter the near edge of the objective. It is thus determined by

4 $$ \varepsilon _{i} = R_{\odot }+ \frac{\left ( r_{d} - r_{a} \right )}{d}\,, $$

where again $R_{\odot}$ is the apparent solar radius (an angle) rather than the actual physical size of the Sun (a length). The narrower the occulted umbral buffer $r_{d}-r_{a}$ can be, the narrower the FOV. Further, for a given required umbral buffer $r_{d}-r_{a}$ around the aperture, larger/longer instruments can image closer to the Sun than smaller/shorter instruments.

The design drivers for the external shaded-truss concept are the structure of the occulter and the design of the support.

The occulter itself must combat Fresnel diffraction of sunlight around the occulting edge. Existing externally-occulted coronagraphs use carefully aligned multidisk designs with tight positional and angular tolerances. We designed an occulter form (Section 3.2) that greatly increases alignment tolerance while still reducing Fresnel diffraction (compared to a disk) and maintaining light weight.

The shaded-truss design supports the occulter on a cantilever truss that must be stiff enough to maintain occulter alignment, while remaining fully within the umbral shadow of the occulter and also obscuring as little of the FOV as practical. We designed a simple hexapod truss built from narrow carbon fiber rods (Section 3.3).

The length of the truss is only loosely constrained by the design. In general, longer trusses perform better: the Fresnel diffraction brightness varies as $\sqrt{\lambda /L\theta }$, where $\lambda $ is wavelength, $L$ is the length between the occulter and objective lens, and $\theta $ is bend angle (inner FOV edge). The mechanical stiffness (first normal-mode frequency) and buckling resistance of the truss constrains the length: occulter mass increases as $L^{3}$ and the first normal-mode frequency of the spring pendulum formed by the truss and occulter therefore decreases as $L^{-2}$. We chose 75 cm to place the first normal mode frequency in the 10 – 20 Hz range with 2 mm diameter carbon-fiber rods and an occulter under 50 g, and found that it met the Fresnel diffraction requirements for this particular instrument.

### Occulter Design

Occulters are limited in effectiveness by Fresnel diffraction, which allows direct sunlight to diffract around the sharp (projected) edge of the occulter and into the optics. The effect of diffraction is reduced by larger instrument size or by larger inner-edge elongation angles, and by multiple bends. Fresnel scattering effects are in general complicated and require careful numerical analysis. However, in simple geometries and approximations, Fresnel diffraction is tractable. The straight razor-edge approximation to Fresnel diffraction can be performed with a 1D integral and requires a cornu-spiral calculation (e.g. Hecht and Zajac, 1974, §10.3.9). In the case of a single plane-wave (collimated beam), the full integral reduces to the Fresnel special functions $S$ and $C$, and may be written:

5 $$ I_{scatter} = \frac{I_{B}}{F^{2}} \left [ \left (C(\theta F) - \sqrt{ \frac{\pi}{8}} \right )^{2} + \left (S(\theta F) - \sqrt{ \frac{\pi}{8}} \right )^{2}\right ] \,, $$

where $I_{scatter}$ is the intensity of light scattered around the occulter from the single collimated source to a detector, $\theta $ is the scattering angle around a nearly-straight section of occulter, $F\equiv \left (\pi d/ \lambda \right )^{0.5}$, $d$ is the length of “throw” from the occulter to the detector, and $\lambda $ is the wavelength of light being considered. For polychromatic or white light, an integral over $\lambda $ is implied. The special functions $S$ and $C$ are defined by

6 $$ S(\alpha )=\int _{0}^{\alpha}\sin \left (u^{2}\right )du $$

and

7 $$ C(\alpha )=\int _{0}^{\alpha}\cos \left (u^{2}\right )du \,, $$

where $\alpha$ is the independent variable of the function and $u$ is a variable of integration. While Equation 5 strictly applies to linear cases, it is appropriate and conservative for estimating the stray light diffracted around the occulter provided that the ray-approximation impact parameter between a given ray at the aperture and the occulter itself is small compared to the radius of the occulter, i.e., for the observed “bright ring” of Fresnel-scattered light observed near the occulter on the image plane (Howard et al., 2008).

For $d=75\text{ cm}$, $\lambda $ over the range 450 – 650 nm, and an apparent occultation diameter of 1.4 R_⊙_, averaging Equation 5 across plane-wave contributions from the extended solar disk and across wavelength yields a total scattering coefficient of $7\times 10^{-4}$, most of which is contained in the bright ring around the occulter. Taking the in-lens scattering coefficient to be of order $10^{-2}$, which is conservative by roughly $10\times $ compared to typical values, this brightness corresponds to a hazy background brightness of roughly $10^{-5}$ B_⊙_ at 2 R_⊙_ in the FOV. During a 90% annular solar eclipse, this brightness level is reduced by another order of magnitude, to $10^{-6}$ B_⊙_, which is comparable to the expected sky brightness at 2 R_⊙_ in Figure 1.

Conventional occulter designs have evolved from single disks to multidisk assemblies, so that multiple Fresnel scattering events are required for light to enter the instrument aperture (e.g. Howard et al., 2008; Dudley et al., 2023). These assemblies can be difficult to manufacture and to align. One way to simplify alignment is to form the occulter from a section of a sphere as in the spaceborne Association of Spacecraft for Polarimetric and Imaging Investigation of the Corona of the Sun (ASPIICS) instrument (Zhukov, 2016) whose free-flying occulter is intended to operate hundreds of meters away from the aperture; this solution works well when the radius of the sphere is long enough to enforce multiple Fresnel scattering events along the surface of the occulter, which is not the case for CATEcor.

We designed the CATEcor occulter to make use of a characteristic of fused deposition modeling (FDM) 3D printing: printed objects are fabricated in layers, which produces microridges along the structure of the final object. A gently curved 3D printed surface therefore approximates a more precisely machined set of edges similar to the edges of a multidisk occulter. We initially considered a spherical envelope similar to that of ASPIICS because spherical surfaces are simple to align. But a spherical FDM occulter with diameter of a few cm would be sufficiently rounded that typically only one of the FDM ridges would interact with the light, and a spherical occulter would therefore behave optically like a single disk.

Multidisk occulters in general have more gently curved envelopes than a similarly sized sphere. The ideal envelope is close to an “ogive”: a figure of revolution of a large circle, about a chord near the perimeter of the circle. The ogive shape allows a constant angular offset between uniformly-spaced disks. To improve the effectiveness of CATEcor occulter, we used an approximate ogive shape with sufficiently long major diameter to allow multiple FDM ridges to interact with the light, thereby approximating the effect of a series of very finely machined disks in a more conventional occulter.

We approximated an ogive form with a prolate circular ellipsoid. This choice was because ellipsoids are simple to create in 3D CAD programs, by stretching a spherical primitive shape. We selected an ellipsoid with minor diameter 37 mm and major diameter 117 mm, i.e., stretched in the prolate direction by a factor of $\sqrt{10}$ compared to a 37 mm diameter sphere. The 37 mm minor diameter arises from the inner FOV angle and the selected length of the truss suspending the occulter (Figure 2). The stretching resulted in a local major radius of curvature of 185 mm at the equator of the ellipsoid, exactly 10 times the minor radius of 18.5 mm. We retained only a small section of the ellipsoid, near its equator; this yielded an approximate truncated-ogive bowed cylinder shape as diagrammed in Figure 3. This provides for a significant length of interaction between the corrugated surface and near-tangent incident light from the solar disk, while also allowing some angular alignment tolerance for the assembly. The truncation was slightly asymmetric about the equator, as shown in Figure 3, to provide a small amount of additional interaction surface to explore the interplay between stray light and aperture size.

The major radius of curvature of the CATEcor occulter is 185 mm at the widest point, and therefore the 0.4 $\mathrm{R}_{\odot}$ angular separation between the solar umbra and the start of the FOV imposes a separation of 340 $\mu$m between the point of tangency of rays from the solar limb and the closest point of tangency of rays that can enter the aperture of the instrument. To enter the aperture, rays from the photosphere must thus traverse between 0.34 and 2.06 mm of the outer envelope of the occulter, to become part of the observed bright ring around the occulter. The shorter distance represents the smallest bending angle of solar rays; this is traversed by rays from the solar limb point closest to the edge of the FOV, to enter the outermost portion of the aperture. The longer distance represents the largest bending angle, which is traversed by the ray from the solar limb point farthest from the edge of the FOV, to enter the outermost portion of the aperture. Points not on the ultimate perimeter of the aperture are, of course, better shielded and require rays to curve around more of the occulter. The 0.34 – 2.06 mm buffer zone on the curved envelope of the surface reduces overall Fresnel scattering by a factor of 10 – 100 compared to a single razor-sharp edge. This establishes a zone from 0.86 mm above the widest point, to 1.81 mm below the widest point, as the “zone of occultation” in ideal geometry. However, other uncertainties require an occulter thicker than the approximately 3 mm this would imply.

Mounting and aligning the occulter is a significant challenge for an instrument that is designed to be reproduced by students, and we therefore designed it with wide angular tolerance; this translates to additional thickness beyond that required for the active occultation zone. Canting the occulter by 1^∘^ moves the point of tangency up by 3.2 mm on one side and down by 3.2 mm on the other, while maintaining the overall occultation properties. Further, the observed head positioning uncertainty of typical FDM 3D printed objects is 50 – 70 $\mu$m; that yields an uncertainty in the occultation zone placement of an additional $\pm 4.0\text{ mm}$. Thus the minimum height of the occulter is dominated by alignment uncertainties: a total of 7.2 mm are required on either side of the band of active occultation, for combined fabrication and alignment tolerances.

FDM printed objects are formed in layers, which form a corrugated surface with small bulges at the center of each layer and small canyons between the layers. The CATEcor occulters are printed at 50 $\mu$m layer thickness, ensuring at least 50 layers across the zone of occultation, and a minimum of 6 layers for any one photospheric ray to bend around. Compared to a fully smooth polished ogive surface, the layering yields a modicum of resistance to particulate contamination, by reducing the effect of invisibly small 50 $\mu $m-sized dust particles in the non-clean-room environment of a remote observing site.

The CATEcor occulters are specifically designed for manipulation of the inner FOV, to explore further occultation if necessary. Therefore they are extended 3 mm farther in the direction of the instrument, providing meaningful additional deeper occultation (with a wider occultation zone) out to 3.2 R_⊙_. The final design is thus a “puck” some 20 mm tall: a truncated ellipsoid (approximating a truncated ogive) with minor radius 18.5 mm and major radius 185 mm, with the widest point (widest cross section, at 37 mm diameter) 1.5 mm above the centerline between top and bottom, and 8.5 mm behind the front (Sun-facing) surface. As implemented with FDM printing, the puck is microcorrugated at the 50 $\mu$m scale.

A through-hole at the center of the occulter permits alignment during assembly, and is blocked in use by black adhesive tape.

The CATEcor occulters also contain 2.5 mm diameter blind holes to mount 2.0 mm o.d. truss rods during assembly. These holes occur in pairs on a radius of 14.5 mm from the centerline, and are tilted by 1.1^∘^, to form the hexapod. The holes are slightly oversized to avoid overconstraining the rods during assembly.

### Shaded-Truss Design

Externally occulted coronagraphs generally use a large baffled “vestibule” to control stray light, with an occulter supported by a rigid pylon (e.g. Brueckner et al., 1995). Instead, CATEcor supports its occulter with a carbon-fiber truss that is directly shaded by the occulter, simplifying stray light control by keeping the support structure out of direct sunlight. The design eschews the vestibule entirely. The optical field of regard is limited by a baffle mounted on the telescope tube, as diagrammed in Figure 2. At a cost of increasing the complexity of the vignetting function of the instrument, this support method provides simplicity, greatly eases alignment, and reduces total instrument mass and complexity.

CATEcor uses the simplest possible fully-constrained truss: six rods mounted between two equilateral triangles of mount points, one on the occulter, and one on the perimeter of the optical aperture. We chose carbon fiber for the rod material, for its ready commercial availability, high strength-to-weight ratio, and stiffness. The rod diameter is 2 mm, chosen to reduce vignetting while supporting a light occulter at 75 cm distance from the mount. The Euler buckling force limit for an unsupported 75 cm long, 2 mm diameter carbon-fiber rod is 4 N, with $10\times $ safety factor; CATEcor uses glue to fix the rod ends, providing angular support and increasing the safety factor by another factor of 4. The occulter mass is approximately 10 g, which imposes a bending force of 2 – 3 N on the truss when extended horizontally, well within the capability of the six 75 cm rods composing the hexapod.

### Aperture Design

To minimize the dark aperture region in Figure 2, the CATEcor aperture is not a complete circle: it is encroached upon by three decks supporting shaded hexapod feet, reducing the diameter of the required dark-shadow region. The aperture and truss are guarded by a thin printed circular baffle (Figure 2) whose leading edges are just inside the umbra of the occulter and just outside the aperture-plane triangle formed by the truss rods. The top few mm of the baffle are just 0.5 mm wide to separate the umbra from penumbra while retaining as much open aperture as possible.

Figure 4 shows the effective unvignetted aperture for three individual points on the sky: the points at 1.5 R_⊙_, 2.0 R_⊙_, and 2.5 R_⊙_, on the planned observing date of 14 October 2023 (apparent solar radius = 16′). The top edge of each colored unvignetted aperture region is defined by the occulter itself; the diagonal “cutouts” at 2 R_⊙_ and 2.5 R_⊙_ are from the truss rods.

The instrument resolution is governed by Fraunhofer diffraction through the effective aperture, and is anisotropic. The radial diffraction limit at 1.5 R_⊙_ is set by the 1.5 mm distance from top to bottom of the sliver of effective aperture, and is roughly 1.5′ – 2′, comparable to human visual acuity. The tangential/lateral diffraction limit is roughly 0.3′ at that distance from the Sun. At 2 R_⊙_, the radial diffraction limit is roughly 0.5′, and the lateral is under 0.1′. Above 2 R_⊙_, the effective resolution is likely to be limited by noise effects rather than seeing or diffraction (DeForest et al., 2018).

CATEcor includes an optical-grade adjustable-iris aperture stop, located just behind the three hexapod decks. The stop is adjustable to explore the trade between inner FOV diameter and stray light with smaller optical apertures.

The 28 mm diameter primary aperture, hexapod supporting decks, and tube/rim baffle are 3D printed as a single piece (Figure 5). The aperture piece supports the hexapod and is supported by a front plate located behind it. Each hexapod deck has two 10 mm deep blind holes, 2.5 mm in diameter, canted at 0.6^∘^, to support the ends of two of the 2 mm diameter hexapod rods. The holes are slightly oversized compared to the rods, to provide alignment play and to allow room for glue to bond the rods rigidly to the aperture piece.

The aperture piece is fixed precisely relative to a supporting front plate, by six radially-aligned rectangular alignment holes in the bottom surface. These mate with rectangular-extrusion alignment tabs in the front plate. The pieces press-fit together, and small finger tabs are provided to mate/demate the pieces. The aperture piece is secured by six M3 fasteners which extend, via through-holes that penetrate the aperture piece and front plate, into captive square nuts in a supporting telescope tube.

A slot in the aperture piece gives tool access to an adjustment lever on an adjustable-iris aperture stop underneath. The slot is 10 mm wide, too narrow for fingers but wide enough to reach in with a small screwdriver or Allen key. The slot is interrupted by a plastic beam to maintain stiffness of the entire part. The top surface is radially beveled to reduce glinting stray light after surface treatment (painting). The slot for adjustable-iris control acts as a light trap in use, and does not noticeably increase stray light or glint.

### Front Plate Design

The aperture piece rests on a front plate interface that accepts and supports an adjustable-iris aperture stop assembly, and in turn rests on a telescope adapter tube. The aperture stop assembly is bolted in place with three M6 through-bolts that mate with captive hex nuts on the underside of the front plate. The caps of the bolts recess into holes in the aperture piece. The bolt holes are slightly oversized to allow adjustment of the lateral positon of the aperture stop during assembly, before the bolts are torqued down. Positional alignment is maintained between the mounted aperture piece and the bolted-on aperture stop, via six extruded alignment features that mate with alignment holes on the underside of the aperture piece. A circular mounting ring, together with a corresponding groove on the underside of the aperture piece, forms a four-bounce “optical maze” to prevent stray light entering the dark space behind the aperture.

### Telescope Tube Extension Design

The telescope tube extension is fabricated with 10 mm thick walls for stiffness, and is secured to the CATE24 telescope tube by six nylon M8 bolts forming a dual triangular friction mount. The nylon M8 bolts are retained by hex nuts held captive by interior hexagonal holes. The front plate and aperture piece are secured to the tube with six steel M3 bolts that engage with six captive square nuts near the top of the piece.

## Fabrication and Integration

All 3D printed parts of CATEcor were printed on a hobbyist 3D FDM printer (PRUSA Mk 3) in black polyethylene terephthalate glycol (PETG) plastic. The FDM settings depended on the part. The telescope tube and front plate were not required to have particularly precise shape and we printed them at high speed with 200 $\mu$m layer thickness and 15% infill. For the aperture piece we used “precision” (lower speed) extrusion settings, with 150 $\mu$m layer thickness, with 30% infill and doubled perimeter wall thickness and top/bottom surface layer count, for rigidity. The occulter required highly precise form and was iterated several times to optimize printer-specific parameters for the cleanest print. We printed it with 50 $\mu$m layer thickness, with external perimeters deposited first to provide the cleanest possible exterior shape, and 30% infill.

The trickiest and longest integration step was assembling the front-end assembly comprising the occulter, hexapod rod truss, and aperture piece. We describe that process here, to illustrate its simplicity and the lack of any special tools required.

**Figure 8:**
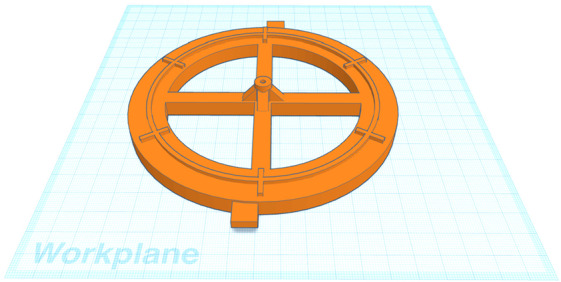
A jig piece used to align the occulter assembly matches the shape of the CATEcor front plate, with rigid support for the seventh alignment rod at the center of the assembly. The central gusseted tube fits into the central hole of the CATEcor aperture piece, to align the aperture piece and occulter while the hexapod truss undergoes assembly and gluing.

To align the occulter we printed a separate jig piece (Figure 8) that accepted a seventh 2.0 mm diameter rod. We placed the jig directly under an alignment hole drilled into a “$2\times 2$” wooden alignment beam that was rigidly mounted 90 cm above the work surface. We aligned the jig piece using a plumb bob, then marked the work surface at the location of the jig piece so that the jig piece could be moved and replaced at the same location.

We secured the rods to the occulter and aperture piece with household 2-hour cure epoxy. After mixing the epoxy, we placed the occulter, upside down, on a clean work surface, and used a bit of rod scrap to pack the six hexapod mount holes in the occulter approximately 25% full of epoxy. We wiped up excess epoxy using a paper towel, wiping radially inward from the edge of the occulter to avoid smearing any epoxy on the active surface.

We mounted an aperture piece on the jig, then threaded the occulter, sunlit side up, onto an uncut (120 cm long) 2 mm rod, threaded the top of the rod upward through the hole in the alignment beam, then threaded the bottom of the rod downward through the aperture piece into the central tube of the jig. Finally, we placed the jig inside the alignment marks on the work surface. This provided lateral positional alignment of approximately ± 2 mm between the occulter and aperture piece, equivalent to angular alignment of $\pm 10^{\prime }$. The occulter central hole fit tightly on the central alignment rod, affording approximately $\pm 20^{ \prime }$ of angular tolerance for an overall $\pm 0.5^{\circ }$ alignment precision compared to the $\pm 1^{\circ }$ design tolerance.

Once the jig, aperture piece, alignment rod, and occulter were placed under the alignment beam, we assembled the hexapod. For each rod, we wiped the ends with an isopropanol-wetted paper towel, waited a few seconds for the ends to dry, then dipped one end approximately 1 cm deep in the epoxy, wiped it on the mixing container to remove excess, carefully inserted the glue-covered end into one of the mount holes in the aperture piece, and finally bowed the rod to insert the clean end into the corresponding prepacked mounting hole in the occulter. During the insertion, we secured the occulter by grasping it between thumb and forefinger, by the sunlit and shaded flat surface (not the active surface). For each rod, we examined the hole ends for excess epoxy and wiped any excess using the end of a 1.5 mm flat-blade screwdriver. We assembled the rods in circular order around the hexagonal assembly. When all rods were inserted at both ends, we visually inspected the occulter for placement, and – holding the rods and not the occulter – we rotated the occulter roughly 45^∘^ to either side of its ideal alignment, to further spread the epoxy in the holes, before aligning the occulter rotation angle.

To set the rotation angle we sighted through the truss, standing along one of the six mirror symmetry planes of the truss assembly. Pairs of rods form vee shapes at the bottom of the truss, and complementary pairs form inverted-vee shapes at the top of the truss. Standing so that one vee on the front side bottom lines up with the central rod should also cause the complementary inverted-vee on the rear side top to line up with the central rod. We twisted the occulter into best visual alignment by sighting along one of the three major symmetry planes and verified alignment by sighting along the other two.

We allowed the epoxy to cure for 8 hours, then removed the central rod from the jig, and removed the aperture–truss–occulter assembly. We verified rigidity by plucking the truss while holding the aperture piece firmly against the work surface, to observe the low oscillation modes and verify the low-amplitude fundamental to be at or above 10 Hz. Because the hexapod is the minimum complete support system, even one loose glue joint has a large effect on the fundamental mode frequency and is obvious.

Based on initial testing (Section 5), we treated the surface to reduce glint. We applied a light coat of commercial Krylon ultra-flat camouflage black spray paint to the entire occulter assembly, holding the spray can some 8 inches from the occulter and lightly “spritzing” the paint. Similar treatment was applied down the length of the truss and in the interior of the aperture assembly, to prevent glint from the finished plastic. Particularly on the occulter itself, and also on the truss, we did not attempt full coverage, just a single very light coat. This was sufficient to intercept any glint coming along the occulter at grazing incidence. We tested both coated and uncoated assemblies and found the stray light from uncoated assemblies to be reduced by 3 × or more in the coated assembly (Section 5).

**Figure 9:**
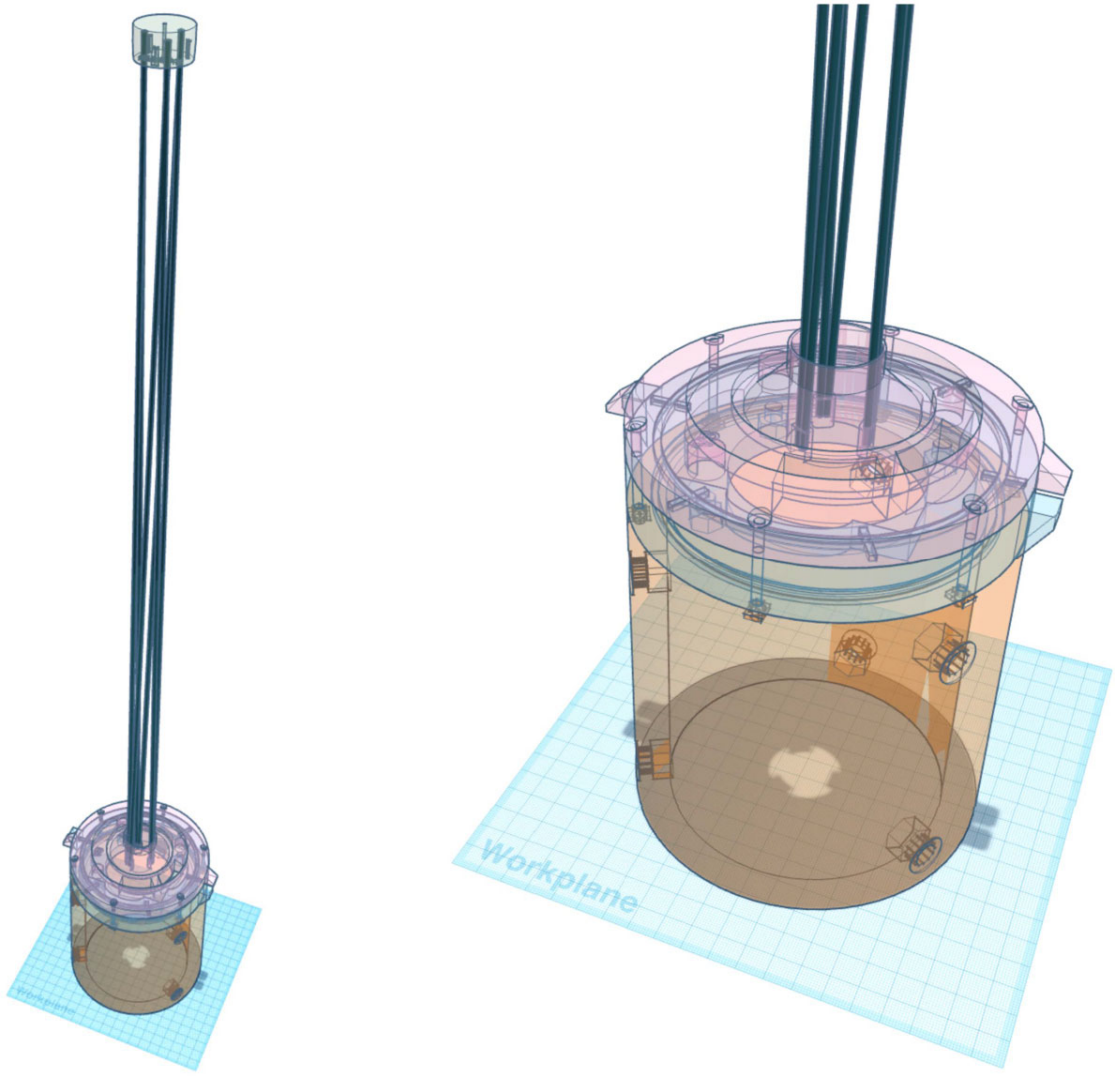
CATEcor adapter assembly drawing shows how the various parts, including occulter, fit together. All parts are 3D printed using fusion deposition modeling at various layer widths, except for the truss rods – which are 2 mm diameter pultruded carbon fiber, cut to 76 cm length.

Assembling the rest of CATEcor is more straightforward (9). We fitted M8 nylon screws with a bolt and washer, then threaded them through the holes in the telescope extension tube and secured them with captive nylon nuts in the interior hexagonal holes. We fitted a commercial iris into the front plate and bolted it in with M6 bolts and captive nuts. We placed the front plate on the telescope interface tube alignment ring. We placed the aperture/occulter assembly on the front plate, so that the ejection tabs on the perimeter approximately lined up, then secured the entire assembly using six M3 bolts through the six perimeter holes in the aperture assembly, into captive square nuts in the telescope extension tube. This resulted in a fully assembled CATEcor adapter assembly as shown in Figure 9.

To observe, we integrated the CATEcor assembly to a CATE24 telescope, on-site at an observing location. We developed a procedure to avoid direct solar exposure through the unprotected telescope onto the detector. We first aligned the telescope and aimed it at the Sun, tracking solar rotation, with a solar filter on the objective lens. One person held a shadow mask above the telescope, casting a shadow onto the objective, while another person removed the solar filter and replaced it with a CATEcor assembly. We stopped down the iris aperture, and iterated a process of briefly removing the shadow mask to observe the shadow of the occulter, then adjusting the nylon set screws to bring the shadow closer to being centered on the aperture and checking alignment again. After 3 – 4 iterations the occulter shadow was centered over the aperture and we removed the shadow mask entirely. During observation, we observed the aperture periodically and used the telescope pointing controls to recenter the solar image and shadow as needed.

## Initial Testing

We constructed an engineering unit of the CATEcor coronagraph front-end assembly, and performed simple testing on it including deployment on a CATE24 telescope on a sunny day.

**Figure 10:**
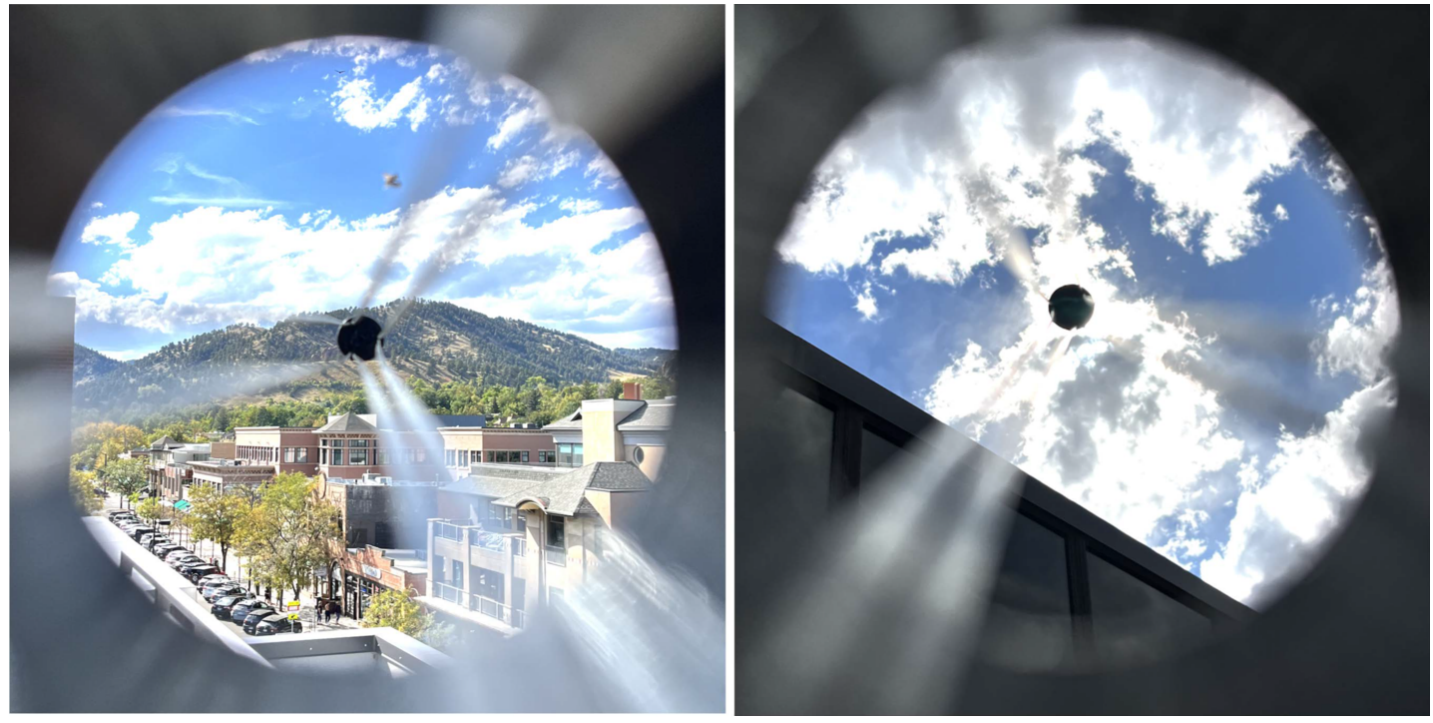
Initial test images taken through the CATEcor engineering unit reveal the geometry of the instrument front-end: (left) the Colorado front range above Boulder, CO; (right) the Sun with light clouds. These images were collected with a cell phone camera held at the aperture at the rear of the assembly. The truss is more strongly visible and the occulter appears slightly larger than in final images, because the cell phone effective aperture is much smaller than the full available aperture at the rear of the assembly.

The aperture–truss–occulter assembly, fully assembled, has a mass well under 1 kg and is readily manipulated by hand. It is easy to sight through the aperture and occulter itself directly with one’s eye or with a small camera. Figure 10 is the result of a crude initial optical test: a photograph of the Colorado Front Range above Boulder, Colorado, taken through a cell phone camera held at the aperture of the assembly; and an exposure of the Sun through light clouds taken in the same circumstance.

The truss structure is more strongly visible in Figure 10 than in scientific images through a CATE24 telescope, because the cell phone aperture is much smaller than the available aperture at the rear of the occulter assembly. The truss takes the appearance of three pairs of “parallel” rods extending to the occulter. The “parallel” rods are actually diverging from the aperture side of the hexapod to the occulter, and the widely spaced rods are converging toward single mount points on the occulter.

**Figure 11:**
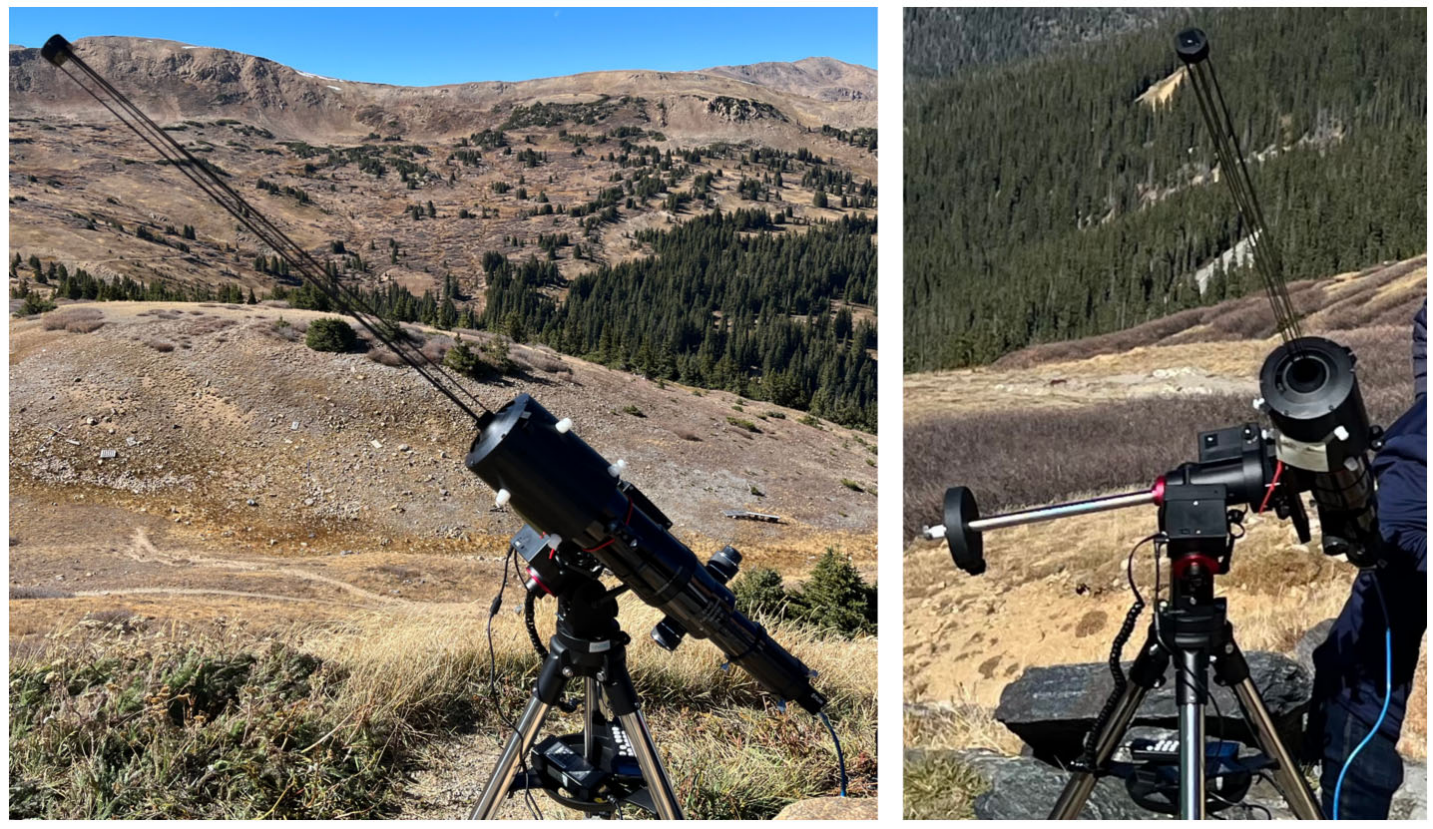
Initial deployment of the CATEcor at Loveland Pass, Colorado, on 6 October 2023, helped establish alignment procedures, demonstrated dark occultation, and revealed some glint around the uncoated occulter. The side view shows the 75 cm distance between the rear of the occulter and the interior of the aperture piece. The front view shows the apparent extreme darkness of the instrument aperture, which is bathed in deep umbral shadow from the occulter.

After full integration, we deployed CATEcor on a clear sunny day at an altitude of 12 000 ft. at Loveland Pass, Colorado, on 6 October 2023, in an interval surrounding noon (roughly 12 pm – 3 pm MDT; noon occurred at 12:54 pm). The fully deployed instrument is shown in Figure 11. Despite a low cloud layer to the north and northeast of the observing site, the sky color at the pass was dark cerulean to cobalt blue with minimal white (Mie-scattered) halo around the Sun, reflecting good “coronal sky” conditions. At the pass we observed some light specks in the air, which we speculated to be high-flying pollen from aspens or high-altitude grasses. Wind levels varied from 5 to 15 knots through the course of the observation, resulting in visible wind shake of the occulter shadow when the occulter was aligned on the Sun. The wind did not visibly shake the telescope itself (see Seaton et al., 2024, for additional description of our initial field test).

**Figure 12:**
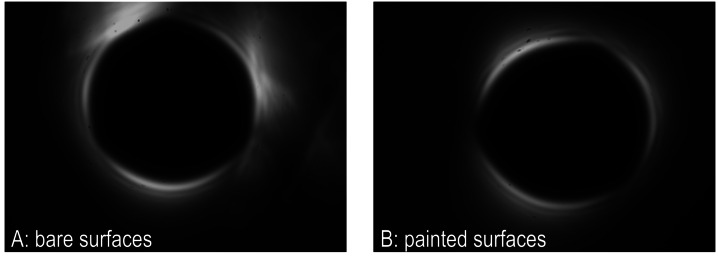
Bare/painted either/or test images with two copies of the CATEcor occulter show the importance of surface treatment to prevent specular reflection and glint. Both images were collected with a single CATE24 telescope and two separate Sun-pointed CATEcor occulters: one bare and one painted. These images are from Loveland Pass, Colorado, on 13 October 2023, and have the same exposure time and scaling. Panel A shows the diffraction/stray-light pattern with bare surfaces. Panel B shows the diffraction/stray-light pattern with painted surfaces. Angular glint features and overall background are greatly reduced with the painted occulter.

Initial images from 6 October 2023 were sufficiently dark that we proceeded to manufacture and integrate several more occulter assemblies, but the images did include features that we surmised to be glint. To half of the new occulters, we applied a flat surface treatment as described in Section 4; on the other half we left the occulter and truss surfaces uncoated. On 13 October 2023 (the day before the eclipse itself), we performed either/or tests in similar conditions by swapping out and realigning the occulter assembly from a fully assembled CATEcor at each of two observing sites for the 14 October 2023 annular/partial eclipse. The two observing sites produced comparable results and revealed that flat black paint reduces glint without appreciably affecting the diffraction pattern around the occulter. Figure 12 shows typical stray light images from the uncoated and coated occulter assemblies in full Sun.

Figure 12, panel B, shows a typical stray light pattern from a coated CATEcor occulter assembly. The familiar diffraction “bright ring” is visible, and is conjugate to an Arago spot (Arago, 1819) formed by the occulter at the entrance aperture of the telescope. The ring is interrupted in three places by vignetting due to the truss holding the occulter. The bright ring has an inner radius of approximately 1.3 R_⊙_, and extends to 5 subsidiary peaks extending to 1.5 R_⊙_. The extent of the subsidiary peaks is a measure of precision in the figure of the occulter. It is a sign both that the signal at this dynamic-range setting is dominated by diffracted light as expected, and also that the occulter and truss work as designed: while present in the FOV, the shaded truss does not significantly contribute to the stray light pattern observed in the final CATEcor coronagraph images.

We used painted CATEcor occulters on CATE24 telescopes at both Loveland Pass, Colorado, and Sandia Peak, New Mexico, to observe the partial or annular (respectively) eclipse of 14 October 2023. Results of that experiment, including initial detection of the solar corona out to 2 R_⊙_ from Sun center, are reported in Seaton et al. (2024).

## Discussion

The CATEcor coronagraph, as built, is best described as a proof-of-concept instrument. We have conceived, designed, produced, and tested a novel class of instrument, a shaded-truss externally occulted coronagraph that takes advantage of the umbral shadow of the external occulter itself to reduce (and effectively eliminate) stray light from the support structure.

### Design Advantages

The shaded-truss concept has several advantages in the coronagraph design space. By separating the functions of stray light control and occulter support, shaded-truss designs greatly reduce the bulk of the coronagraph front-end. In the CATEcor design, only a very small baffle isolates the aperture itself from the bright sunlight impinging on the instrument, essentially reducing the front-end stray light control structure to a minimalist support and a very lightweight occulting body. Further, because the front-end support is very light, it is feasible to suspend an external occulter at surprisingly large distances from the aperture, improving performance compared to designs with a stray-light control structure (i.e., vestibule) surrounding the occulter and a conventional support pylon.

These advantages of the shaded-truss approach are sufficiently great that we were able to conceive, design, manufacture, integrate, and test a coronagraph front-end in under six weeks, using freeware CAD tools, hobbyist parts, and a consumer-grade FDM 3D printer. Despite the preposterously simple design, fabrication, and integration approach – which are accessible to any hobbyist with access to a “makerspace” workshop and a good hardware store – the resulting coronagraph is demonstrated to be cleanly diffraction limited and readily deployable in combination with common amateur-astronomy equipment. This, in turn, implies that shaded-truss coronagraphs have potential to be important tools either for educational and amateur coronal viewing, or for quantitative scientific work. We also demonstrated this particular design by observing the Sun’s corona at the annular (partial) eclipse of 14 October 2023 (Seaton et al., 2024).

Compared to conventional externally occulted designs, shaded-truss coronagraphs can bring the occulter farther from the aperture, reducing the limitations of external occultation. The ideal external occulter is infinitely far from the aperture and infinitely large, as exemplified by the Moon during a total solar eclipse. Existing externally occulted designs use separation distances well under a meter, largely because it is infeasible, in a deployable or spaceborne instrument, to build a large combined structural and stray-light-controlling structure in front of the optics. This limits the sharpness of the partially-vignetted zone around the Sun, and therefore the inner radius of the FOV of externally occulted coronagraphs. By permitting a long cantilever distance between the aperture and occulter, shaded-truss designs better approximate the ideal conditions of a total solar eclipse, lessening the primary disadvantage of external occultation.

Conventional externally occulted coronagraphs have strictly limited fields of view, which are determined by the diameter of the aperture in the leading structure. By divorcing the functions of structure and stray-light control, a shaded-truss coronagraph affords a much broader FOV. The CATEcor outer FOV was limited by the CATE24 telescope itself at a few solar radii; but the occulting assembly, which was not particularly optimized for FOV, could nevertheless admit images beyond 40 R_⊙_ in all directions (Figure 10). This permits a broader design space for future instruments that could in principle cover from a few tenths of a solar radius to many degrees from the Sun in a single field of view.

### Lessons Learned

Several immediate design improvements are apparent from this proof-of-concept study.

The occulter itself uses the corrugation inherent to 3D printing to force multiple diffractive scatters for light to enter the primary aperture. We found, on deployment, that this fine corrugation was not sufficiently deep to prevent dust and other forms of contamination from “spoiling” the cleanliness of the active surface of the occulter. On deployment, we noticed that small bright quasiglints could be seen around the perimeter of the occulter. Close inspection showed that these glints were dust particles, fibers, and other contaminants that landed on the surface during observation and extended into the bright sunlight around the occulter itself. Designing and printing a more deeply, explicitly corrugated surface would greatly reduce the effect of this type of contamination, while not affecting strongly the other properties of the occulter. Likewise, blowing off dust and lint with filtered, clean air or nitrogen just before acquiring data may improve future observations.

Aligning the occulter and telescope to the Sun was difficult. Understanding the alignment post facto, during analysis, was similarly difficult, since the Sun was (by design) not visible behind the occulter, and there were no other celestial references visible in the FOV. We are developing a design adaptation that would allow us to track the position of the Sun relative to the occulter, to solve this problem in future iterations.

The stiffness of the final occulter assembly was limited by the stiffness of the occulter rods. We used 2 mm diameter rods in CATEcor, and in conjunction with the chosen 75 cm length of the truss, this choice imposed a roughly 10 – 15 Hz fundamental frequency for small perturbations; the frequency was determined by the stiffness of the rods themselves rather than of the truss as a whole. At this modest level of stiffness, wind shake was a significant issue even in light breezes. Subsequent designs could use a combination of thicker rods and/or a mid-rod stiffener bracket to raise the stiffness, ideally into at least the 20 – 30 Hz range.

While FDM 3D printing is a convenient process for prototyping, it is not required for implementing a coronagraph of this general type. Other fabrication methods, including (additive) resin printing and (subtractive) conventional machining, and other materials, including metals and stiffer plastics than the PETG used, provide much more precision and performance, and would improve both the diffractive/optical performance and stiffness of this demonstration design.

The offset between the occulter and aperture is not strictly limited to 75 cm as in the CATEcor design. Commercial circular-cross-section carbon fiber rods afford excellent strength-to-mass ratio and stiffness, but improved truss designs and scientific-grade materials afford yet greater strength. Lengthening the aperture-occulter distance improves performance by reducing the inner diameter of the FOV, while also reducing the Fresnel diffraction around the occulter. The truss design imposes a necessary tradeoff between complexity of vignetting function and rigidity of the occulting structure; CATEcor is a first exploratory cut at this novel design space, and is far from optimized.

## Conclusions

CATEcor is a proof-of-concept of a new type of instrument – a shaded-truss externally-occulted coronagraph. We designed it specifically to match the conditions of the 14 October 2023 annular eclipse, but the concept has applications beyond our initial deployment. In particular, because CATEcor was designed entirely with open-source CAD tools and implemented with materials and procedures available to amateur astronomers and advanced students, it demonstrates the feasibility of “from scratch” observations of the solar corona for non-scientists, students, and amateur astronomers. Further, CATEcor opens a new design space of shaded-truss coronagraphs, with the potential to offer better performance and broader fields of view than conventional designs.

## Data Availability

No datasets were generated or analysed during the current study.

## Footnotes

1 The shaded-truss design is the subject of a patent application by Southwest Research Institute. First public disclosure was 6 October 2023. Contact the authors for more information.

## Materials Availability

The mechanical design is intended to be accessible to the public. Printable .stl files are published on Thingiverse (DeForest, 2023).
